# Reactive Oxygen Species in Plants: Metabolism, Signaling, and Oxidative Modifications

**DOI:** 10.3390/antiox14060617

**Published:** 2025-05-22

**Authors:** Chao Zheng, Jian-Ping Chen, Xiao-Wei Wang, Ping Li

**Affiliations:** 1 State Key Laboratory for Quality and Safety of Agro-Products, Key Laboratory of Biotechnology in Plant Protection of MARA, Zhejiang Key Laboratory of Green Plant Protection, Institute of Plant Virology, Ningbo University, Ningbo 315211, China; 2 Ministry of Agriculture Key Lab of Molecular Biology of Crop Pathogens and Insects, Institute of Insect Sciences, Zhejiang University, Hangzhou 310058, China

**Keywords:** reactive oxygen species, oxidative modifications, signal transduction

## Abstract

Reactive oxygen species (ROS) serve as crucial signaling molecules that facilitate the interactions between plants and environmental stimuli, thereby influencing a wide range of physiological and biochemical processes, such as vegetative apex development and organ morphogenesis. In response to environmental stresses, plants enhance ROS production to initiate a robust protective response. To manage excessive ROS levels, plants have developed a sophisticated antioxidative defense system comprising both enzymatic and non-enzymatic components, which work synergistically to scavenge ROS and alleviate ROS-induced deleterious effects on biomolecules. This review provides a comprehensive overview of ROS metabolism, signaling transduction pathways, and their implications for the oxidative modification of nucleic acids, lipids, and proteins within plant cells.

## 1. Introduction

Reactive oxygen species (ROS) encompass a group of chemically reactive oxygen (O_2_)-containing molecules that play an important role in plant responses to adverse environmental conditions [1]. ROS are by-products of aerobic metabolism, affecting numerous aspects of the plant life cycle and environmental response. Under normal growth conditions, the plant antioxidant system maintains a dynamic balance between ROS production and scavenging [2]. Nevertheless, under biotic and abiotic stresses, the antioxidant mechanism can be compromised, disrupting the balance between ROS generation and elimination, ultimately leading to excessive ROS accumulation and an oxidative burst [3,4]. In the context of plant–environment interactions, plants generate a certain amount of ROS involved in regulation of various processes including insect [5] and pathogen defense [6], plant programmed cell death (PCD) [7], and stomatal behavior [8].

Maintenance of genome integrity is crucial for all living organisms, as it is essential for proper development and for faithful transmission of the genetic information from one generation to the next [9]. However, organisms are continually subjected to DNA lesions. The major sources of mutations arise from stress conditions, such as UV, heat, or drought, during which plants can produce ROS [10]. Excessive accumulation of ROS can result in DNA damage, including single-strand DNA breaks (SSBs) and double-strand DNA breaks (DSBs), as well as intrinsic DNA damage [11,12]. This damage can compromise plant genome stability. Pretreatment with the radical-scavenging compound N-acetyl cysteine has been shown to reduce the homologous recombination frequencies induced by oxidative stress-causing agents, such as rose Bengal (RB), paraquat (PQ), and amino-triazole (ATZ) [13]. *Arabidopsis* RUG3 and ATM co-regulate mitochondrial ROS (mROS) accumulation in response to methyl methanesulfonate (MMS) treatment. The overaccumulation of H_2_O_2_ in *atm-2* and *rug3-1* plants triggers a DNA damage response [14]. In conclusion, ROS may function as a toxin to organisms.

Despite the presumed toxicity of ROS, ROS are found to acting as signaling molecules interplaying with other signaling pathways, such as calcium ion (Ca^2+^) signaling, MAPK cascades, NO signaling, and phytohormones, to modulate stress responses and plant growth [15,16]. In addition, ROS can react with proteins and DNA or RNA. Protein oxidative modifications can be categorized as irreversible modifications, such as protein carbonyls and 3-nitrotyrosine, and reversible modifications [17]. Reversible modifications often involve cysteine residues, including s-sulfenylation [18], s-nitrosylation [19], s-glutathionylation [20], and disulfide bond formation [21]. ROS-induced oxidative damage can also lead to the formation of oxidized guanine nucleosides, such as 8-oxo-7,8-dihydro-2-deoxyguanosine (8-oxo-dG) from DNA and 8-oxo-7,8-dihydroguanosine (8-oxoG) from RNA [22].

Recent advancements in molecular biology and genetic technologies have significantly enhanced our understanding of the mechanisms by which ROS function in plant–environment interactions [23,24,25]. This article aims to review the mechanisms of plant ROS production and scavenging, their sensing, and implications in the oxidative modification of plant nucleic acids and proteins.

## 2. ROS Metabolism

### 2.1. Types of ROS

ROS include singlet oxygen (^1^O_2_), triplet oxygen (^3^O_2_), superoxide anion (O_2_^•−^), hydrogen peroxide (H_2_O_2_), hydroxyl radical (^•^OH), and various forms of peroxides [26]. ^1^O_2_ is an excited-state form of O_2_, generated through the interaction of O₂ in photosystem II (PSII) with ^3^O_2_-excited chlorophyll. The excitation energy is transferred to carotenoids (Cars), leading to the formation of ^3^O_2_ and the active state of carotenoids 3Cars*. Subsequently, 3Cars* decay to the ground state through two ways, namely radiative transition and non-radiative transition, releasing the energy in the form of heat [27]. Although ^1^O_2_ has a short lifespan and is extremely unstable in cells, its production can significantly impact photosynthesis [28]. O_2_^•−^ is the precursor of various ROS due to its instability, and it can be generated by the photosynthetic electron-transport chain (in chloroplasts), the mitochondrial respiratory electron-transport chain (in mitochondria), and the xanthine oxidase system (in peroxisomes) [29]. Subsequently, O_2_^•−^ can be converted into H_2_O_2_ by superoxide dismutase (SOD), with H_2_O_2_ acting as an important signal component in plants [30]. ^•^OH is generated when the O-O double bond in H_2_O_2_ is broken. This radical is highly reactive and can interact with various biological molecules, oxidizing cell wall polysaccharides, resulting in cell wall loosening and DNA single-strand breaks [31].

### 2.2. Generation of ROS

**Figure 1:**
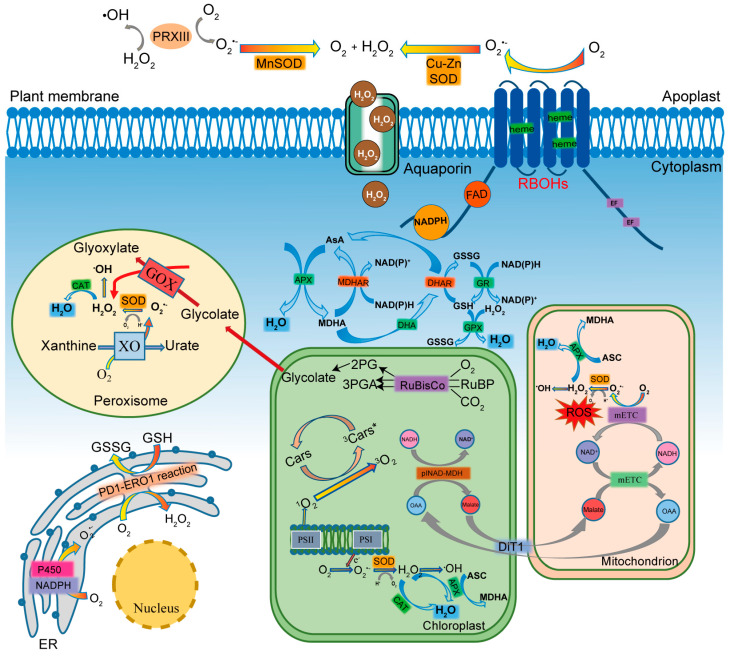
ROS production and scavenging in plant cells. Extracellular ROS are generated by NADPH oxidase on the plasma membrane, which utilizes NADPH as the electron donor to transfer electrons to O_2_ in the apoplast, resulting in the generation of superoxide anion (O_2_^•−^). Subsequently, the O_2_^•−^ undergo a disproportionation reaction and are converted into hydrogen peroxide (H_2_O_2_). Apoplastic class III peroxidases are involved in low-temperature-induced and microbe-associated molecular pattern (MAMP)-induced extracellular H_2_O_2_ production. H_2_O_2_ enters the cytoplasm through aquaporins. Inside the cytoplasm, H_2_O_2_ is detoxified by ascorbate peroxidase (APX), using ascorbate (AsA) as an electron donor. During this process, AsA is oxidized to monodehydroascorbate (MDHA), which is subsequently converted to dehydroascorbate (DHA). DHA is then recycled and regenerated through a series of enzymatic reactions involving monodehydroascorbate reductase (MDHAR), dehydroascorbate reductase (DHAR), glutathione reductase (GR), and the non-enzymatic antioxidant glutathione. Intracellular ROS are generated in organelles such as chloroplasts, mitochondria, peroxisomes, and the endoplasmic reticulum. In chloroplasts, the excited triplet state chlorophyll (PSII) can produce singlet oxygen (^1^O_2_), while the reactions occurring in PSI also promote the generation of O_2_^•−^. The generation of ROS in mitochondria is primarily due to the electron leakage in the mitochondrial electron transport chain. The O_2_^•−^ produced by chloroplasts and mitochondria are rapidly converted into H_2_O_2_ by superoxide dismutase (SOD). GOX, glycolate oxidase; XO, xanthine oxidase. Peroxisomes and glyoxysomes produce a large amount of H_2_O_2_ during the processes of photorespiration and fatty acid oxidation, respectively, and these H_2_O_2_ will be rapidly scavenged by catalase (CAT). Abbreviations: GSH, reduced glutathione; GSSG, oxidized glutathione; PD1-ERO1 reaction, protein disulfide isomerase-endoplasmic reticulum oxidase 1 reaction; P450, cytochrome P450; GPX, glutathione peroxidase; FAD, flavin adenine dinucleotide; RBOHs, respiratory burst oxidase homologs; 2PG, 2-phosphoglyceric acid; 3PGA, 3-phosphoglyceric acid; RuBP, ribulose-1,5-bisphosphate; RuBisCo, ribulose-1,5-bisphosphate carboxylase/oxygenase; Cars, carotenoids; OAA, oxaloacetic acid; PSII, photosystem II; PSI, photosystem I; ASC, ascorbate; mETC, mitochondrial electron-transport chain.

Based on their sites of production, ROS can be classified into intracellular ROS and extracellular ROS (Figure 1). Extracellular ROS, commonly referred to as (e)H_2_O_2_, originate from at least two distinct sources: cell wall peroxidases and plasma membrane NADPH oxidases (NOXs). In *Arabidopsis*, apoplastic class III peroxidases PRX62/PRX69 and PRX33/PRX34 are involved in low-temperature-induced and microbe-associated molecular pattern (MAMP)-induced extracellular H_2_O_2_ production, respectively [32,33]. Additionally, NOXs, also known as respiratory burst oxidase homologs (RBOHs), play a significant role in extracellular ROS production in plants. The plant RBOH proteins contain six conserved transmembrane helices, two heme groups, a C-terminal hydrophilic domain and an N-terminal domain [34]. The N-terminal domain contains two EF-hand structures responsible for calcium binding [35,36], and the C-terminal domain contains FAD and NADPH binding sites [37]. In *Arabidopsis*, the mechanism for RBOH-mediated ROS production has been well-studied. The receptor-like cytoplasmic kinases (RLCKs) and calcium-dependent protein kinases activate RBOH-mediated ROS accumulation by interacting and phosphorylating RBOHs [38,39,40,41,42,43]. Phosphatidic acid (PA) binding [44,45], sulfenylation of cysteine residues [46,47], and endocytosis of RBOHs [48,49] are also crucial for ROS production.

Chloroplasts are the major sources for intracellular ROS [50,51]. There are two basic mechanisms by which ROS are generated in chloroplasts. The first mechanism involves the transfer of an excited electron spin state from chlorophyll to O_2_ to form the highly reactive ^1^O_2_. When light energy is absorbed, one electron is ejected from an electron pair in the chlorophyll molecule in the ground state (S_0_), promoting it to a higher energy state (S_2_). As this electron loses energy, it transitions to a lower energetic state (triplet, T) which is easily populated from the light-induced singlet excited state. Transfer of energy from triplet state chlorophyll to the O_2_ produces singlet oxygen (^1^O_2_) [52]. Secondly, electrons can also be transferred directly to O_2_, leading to the formation of O_2_^•−^. Electron-transport chains (ETCs) in photosystem I (PSI) are the predominant source of ROS in chloroplasts. The Mehler reaction in PSI facilitates a part of the electron flow from ferredoxin to O_2_, resulting in the generation of O_2_^•−^ [53]. Electron leakage to O_2_ may also occur from 2Fe-2S and 4Fe-4S clusters in the ETC of PSI. O_2_^•−^ can also be generated within PSII [54]. Furthermore, O_2_^•−^ are rapidly converted to H_2_O_2_ by the action of the thylakoid O_2_^•−^ dismutases (SODs). The mitochondrial electron-transport chain (mtETC) is another major source of intracellular ROS. Electrons from flavin, metal centers, and quinones in complex I (NADH dehydrogenase) and complex III (cytochrome b/c1 complex) are transferred to O_2_, which is reduced to O_2_^•−^ [55]. The superoxide radical anion has a very short half-life in the mitochondria, where superoxide radical anion undergoes dismutation by manganese superoxide dismutase [56]. Additionally, chloroplasts and mitochondria can jointly regulate the production of ROS. Chloroplast-derived NADH can be used to produce malate, which is then exported to mitochondria, where NADH is oxidized and regenerated. The oxidation of NADH leads to the generation of mitochondrial ROS [29,57].

Peroxisomes are significant sources of ROS, such as H_2_O_2_, O_2_^•−^, and ^•^OH, which are mainly produced in different metabolic pathways, including fatty acid β-oxidation, photorespiration reaction, enzymatic reaction of flavin oxidases, nucleic acid and polyamine catabolism, and ureide metabolism [58].

### 2.3. Scavenging of ROS

To ensure normal growth, intracellular levels of ROS in plants must be finely regulated to maintain cellular homeostasis and prevent oxidative stress. The ROS-scavenging mechanisms in plants primarily consist of both enzymatic and non-enzymatic antioxidant defense systems, which function synergistically to alleviate the harmful effects of excess ROS (Figure 1).

Enzymatic antioxidants include a range of specific enzymes that catalyze reactions to neutralize ROS, thereby protecting cellular components from oxidative damage, with the key players being SOD, ascorbate peroxidase (APX), catalase (CAT), glutathione peroxidase (GPX), and peroxidase (POD) [59]. SODs are classified into three categories based on the metal ions bound to their active sites: Mn-SOD, Fe-SOD, and Cu/Zn-SOD. These enzymes catalyze the reduction reaction of O_2_^•−^, facilitating its conversion into H_2_O_2_ in plant cells [60]. Subsequently, APX reduces H_2_O_2_ into H_2_O using ascorbate as an electron donor [29]. Another two enzymes, CAT and POD, abundant proteins in plant peroxisomes, decompose H_2_O_2_ into H_2_O and O_2_ [61,62]. Additionally, GPX, a thiol-based peroxidase featuring cysteine at its active site, can also convert H_2_O_2_ into H_2_O and O_2_. However, its ROS-scavenging ability is comparatively weaker than that of other antioxidant enzymes, such as CAT or APX [63].

The non-enzymatic defense system of plants refers to the scavenging of ROS through a variety of non-enzymatic substances. Non-enzymatic antioxidants can be further categorized into sulfhydryl compounds (such as glutathione (GSH)), water-soluble vitamin derivatives (such as ascorbic acid (AsA)), iso-prenoid derivatives (such as carotenoids), and phenolic compounds (including flavonoids and other polyphenols). These antioxidants collectively capture and neutralize ROS, thereby establishing a robust protective shield against oxidative stress and safeguarding cellular components from damage [64]. The reduced GSH is particularly necessary in plants, as it effectively scavenges the excessive ROS generated during cell metabolism and helps mitigate damage caused by membrane lipid peroxidation [65]. AsA, a water-soluble organic small molecule widely distributed in plants, has the ability to scavenge ^1^O_2_, O_2_^•−^, and ^•^OH and regenerates tocopherol by donating its electron. Carotenoids, a group of pigments in plants, have the capacity to quench ^1^O_2_ as well as triplet chlorophylls, a special excited state of chlorophyll molecules [66]. Flavonoids, natural substances synthesized by plants, are the ideal scavengers of H_2_O_2_ due to their favorable reduction potentials relative to alkyl peroxyl radicals [67]. In addition, polyphenols also exhibit relatively strong antioxidant capacity and can scavenge ROS, further assisting plants in coping with oxidation-related challenges [68].

## 3. ROS Signaling Pathways

In this review, we discuss the functions of ROS as signaling molecules, highlighting the mechanisms by which ROS are sensed by cells and the processes that govern their transport both within and outside of cells.

### 3.1. ROS Sensing

Cells must be capable of precisely deciphering ROS signals based on the type of ROS, their subcellular localization, and the timing of their production. Various mechanisms have been proposed for ROS perception in plants. One such mechanism involves the plasma-membrane-localized leucine-rich-repeat receptor kinase (LRR) known as hydrogen-peroxide-induced Ca^2+^ increases 1 (HPCA1), which mediates eH_2_O_2_-induced activation of Ca^2+^ by oxidizing the extracellular Cys residues of HPCA1 [69]. Plant quiescin sulfhydryl oxidase homolog (QSOX1) is also recognized as a H_2_O_2_ sensor that interacts with S-nitrosoglutathione reductase GSNOR1 to facilitate redox regulation, contributing plant immunity against *Pst* DC3000 (*avrRpt2*) [70]. In contrast, the endogenous H_2_O_2_ is sensed by the cytosolic thiol peroxidase PRXIIB via oxidation at Cys51, resulting in the inhibition of the ABI2 phosphatase activity [71]. Unlike *Arabidopsis* PRXIIB and HPCA1/QSOX1, which detect cytosolic and extracellular H_2_O_2_, respectively, rice basic/helix–loop–helix transcription factor bHLH25 directly senses H_2_O_2_ in the nucleus and then undergoes oxidation at Met256 to repress miR397b expression and activate lignin biosynthesis [72].

### 3.2. ROS Transport

The short lifetimes of superoxide, the hydroxyl radical, and singlet oxygen make them unlikely candidates to diffuse over appreciable distances within the cell. In contrast, H_2_O_2_ is one more stable ROS type with a relatively long half-life. This feature allows H_2_O_2_ to function as an important redox signaling molecule that can be transported across the plasma membrane and between cells, thereby inducing local and systemic acquired resistance [6,73].

The transport of H_2_O_2_ may be regulated by aquaporin proteins. Fluorescence assays with an intracellular ROS-sensitive fluorescent dye have indicated that *Arabidopsis* tonoplast intrinsic protein (TIP) 1;1 and TIP1;2 promote the transport of H_2_O_2_ in yeast [74,75]. Subsequent investigations revealed that both plasma-membrane-localized aquaporins AtPIP2;1/AtPIP2;4, as well as the tonoplast-intrinsic AtNIP1.2, are capable of conducting H_2_O_2_ in yeast cells [76]. Further studies revealed that aquaporins from the AtPIP2 subfamily, specifically AtPIP2;2, AtPIP2;4, AtPIP2;5, and AtPIP2;7, are essential for H_2_O_2_ conduction in yeast, while aquaporins from the AtPIP1 subfamily are not required for this process [77]. However, another study has shown that AtPIP1;4 is able to mediate the translocation of externally applied H_2_O_2_ into the cytoplasm of yeast (*Saccharomyces cerevisiae*) cells, which can also facilitate the transport of H_2_O_2_ across plasma membranes and mediate the movement of H_2_O_2_ from the apoplast to the cytoplasm in plants [78]. *Atpip2;1* mutants show decreased intracellular ROS accumulation and impaired ABA- and PAMP-induced stomatal closure, suggesting it has a role in both water and H_2_O_2_ transport [79]. Aquaporins are also responsible for the diffusion of chloroplast ROS. Treatment with acetazolamide, the inhibitor of aquaporins, suppresses the intensity of resorufin fluorescence outside the chloroplasts [80]. Additionally, *Arabidopsis* NODULIN 26-LIKE INTRINSIC PROTEIN 1; 1 (NIP1;1) contributes plant response to H_2_O_2_ [81].

Interestingly, the activity of aquaporins is regulated by phosphorylation. For example, the transport of H_2_O_2_ mediated by HvPIP2;5 is dependent on the phosphorylation of Ser126. A mutation that replaces Ser126 with Ala has been shown to restore the growth of Δskn7 cells on H_2_O_2_-containing medium. Consistent with this finding, flg22 treatment induces the phosphorylation of OsPIP2;2 Ser125 and AtPIP2;1 Ser121 sites, enhancing their ability to transport H_2_O_2_ [79,82,83]. While various phosphorylation sites at the N-terminal and C-terminal tails of aquaporins have been identified [84], their roles in plant H_2_O_2_ transport are largely unexplored. In addition, the Lys3 and Glu6 sites at the N-terminal tail of AtPIP2;1 are methylated by SDG7 and OMTF3, respectively [85,86]; however, the functional implications of these modifications on H_2_O_2_ diffusion have yet to be investigated. Moreover, the distribution and permeability of aquaporins appear very dynamic, which could influence H_2_O_2_ signaling. For instance, treatment with 0.5 mM H_2_O_2_ induces a significant depletion in plasma membrane (PM) fractions of several abundant PIP homologs and triggers AtPIP2;1 accumulation in the late endosomal compartments [87]. Despite these insights, the transporter of H_2_O_2_ for long-distance transport remains unknown.

## 4. ROS-Mediated Oxidative Modifications

O_2_, as the precursor of all ROS, can be converted into ROS through various processes that impact plant metabolism across different cellular organelles, thereby influencing the plant’s response to both abiotic and biotic stresses. For a more comprehensive understanding of the functions of ROS in plants, readers are encouraged to refer to several excellent reviews on the topic [88,89,90,91]. In this section, we will focus specifically on the role of ROS in the oxidation of intracellular components, particularly nucleic acids, lipids, and proteins, with a special emphasis on proteins.

### 4.1. Nucleic Acids

**Figure 2:**
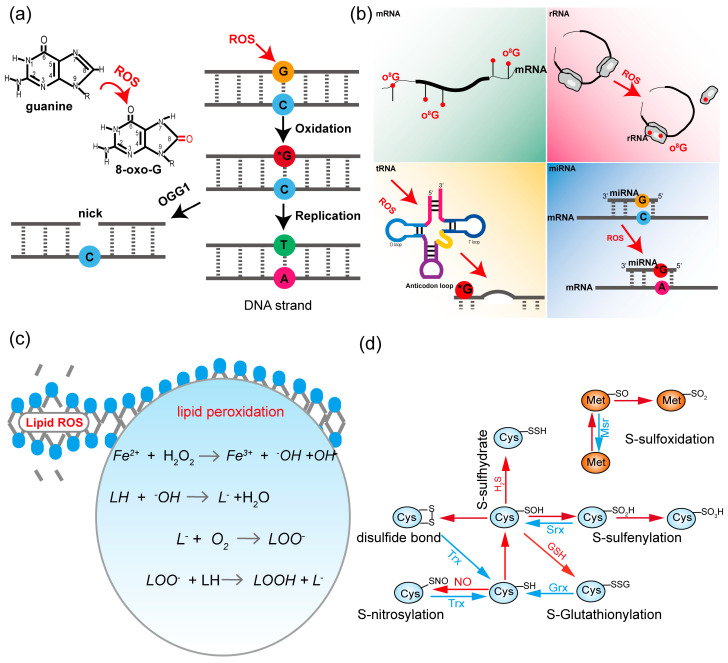
ROS-mediated oxidative modifications. (**a**) ROS attacks the 8th carbon of the guanine base in DNA to form 8-oxo-G and can be recognized and hydrolyzed by OGG1; (**b**) ROS can oxidize G in RNA (mRNA, rRNA, tRNA, and miRNA) molecules to o^8^G; (**c**) ROS can initiate intense lipid peroxidation reactions, ROS will attack the lipid molecules in the cell membrane; (**d**) ROS can oxidize the amino acid residues (Cys and Met) in proteins (bottom right). Abbreviations: 8-oxo-G, 8-oxo-7,8-dihydroguanine; OGG1, DNA glycosylase; mRNA, messenger RNA; o^8^G, 8-oxoguanine; rRNA, ribosomal RNA; tRNA, transfer RNA; D loop, dihydrouridine loop; T loop, TψC loop; miRNA, microRNA; *G, Guanine is attacked by ROS to o8G;LOOH, lipid hydroperoxide; Met, methionine; Msr, methionine sulfoxide reductase; Cys, cysteine; Trx, thioredoxin; Grx, glutaredoxin; Srx, sulfurtransferase; H_2_S, hydrogen sulfide.

ROS exhibit high reactivity and are capable of triggering diverse forms of DNA oxidative modifications. ROS-mediated oxidative damage results in DNA strand breaks and base oxidation (Figure 2a). These alterations severely damage the DNA structure, and are considered as the most serious ROS-induced cellular modifications [92]. The underlying mechanisms and measurement of oxidative damage to DNA have been comprehensively reviewed previously [93,94,95]. In addition to affecting DNA, ROS can also oxidize guanine (G) in various RNA molecules, including mRNA, rRNA, tRNA, and miRNA, converting it to 8-oxoguanine (o^8^G) (Figure 2b). The presence of o^8^G at positions 3 and 4 of miR-124 and o^8^G at position 7 of miR-1 is associated with tumor development and cardiac hypertrophy [96,97]. Targeted mRNA oxidation has also been observed in plants. Targeted mRNA oxidation during dry after-ripening of dormant seeds may govern cell signaling toward germination in the early steps of seed imbibition [98]. ^1^O_2_-mediated RNA 8-oxoG affects the rate of protein synthesis [99]. However, the role of this modification in plant development and responses to environmental stimulus remain largely unexplored in plants.

Additionally, ROS play a role in other epigenetic processes, such as DNA methylation, histone modification, and chromatin structure [100]. Application of 2,2′-azobis (2-amidinopropane) dihydrochloride, a generator of free radicals, in *Pisum sativum* suspension culture clearly decreases the global DNA methylation levels [101]. Overproduction of ROS in tobacco BY-2 suspension cells after treatment with naphthoquinone juglone causes hypomethylation of DNA [102]. DNA methylation induced by heavy metals manganese (Mn) and cadmium (Cd) partially depends on ROS [103]. Cell redox directs different levels of DNA methylation and histone acetylation [104]. Taken together, ROS may influence epigenetic regulation through various mechanisms.

### 4.2. Lipids

In plants, ROS can initiate intense lipid peroxidation reactions. Highly reactive ROS molecules target the lipid molecules in the cell membrane, especially the unsaturated fatty acid moieties [105]. Most of the lipid peroxidation reactions occurring in green plant tissues are initiated by ^1^O_2_. ^1^O_2_ is incorporated into plastid membranes and promotes lipid hydroperoxide (LOOH). H_2_O_2_ is prone to iron-catalyzed degradation to produce ^•^OH and O_2_^•−^. ^•^OH can abstract hydrogen from fatty acids that leads to the formation of lipid radicals (L^•^). Subsequently, L^•^ react with O_2_ and LOO^•^ to produce lipid peroxides (Figure 2c) [106,107]. Once lipid peroxidation is initiated, the structure of lipid molecules is altered, disrupting the stability of the lipid bilayer and damaging the cell membrane structure. This damage to the cell membrane structure affects the fluidity, permeability, and signal transduction processes of the cell membrane [108]. As a consequence, the activity of membrane proteins, the efficiency of substance transport, and cell–cell interactions are impacted. This not only affects the functionality of membrane proteins but may also hinder the absorption of nutrients and the excretion of metabolic waste by cells, ultimately compromising overall cellular health and function.

### 4.3. Proteins

#### 4.3.1. ROS-Mediated Oxidative Modifications

ROS-mediated oxidative modifications of proteins at cysteine (Cys) or methionine (Met) residues have a profound impact on protein structure and function, further affecting protein activity, stability, and interactions. The oxidation of Cys has been extensively studied, revealing that its different oxidation states can affect various proteins. Common oxidative modifications of Cys include the formation of disulfide bonds [109], sulfenic acid [110], sulfinic acid [111], sulfonic acid [112], S-glutathionylation [113], S-nitrosylation [114], and S-thiolation [115] (Figure 2d). These modifications can alter the redox state of proteins and modulate their activity and interactions with other molecules (Table 1). In *Arabidopsis*, both NPR1 and its interactor TGA1 are subjected to S-nitrosylation at Cys residues. S-nitrosylation of NPR1 not only stimulates its nuclear import but also promotes its oligomerization. S-nitrosylation of TGA1 enhances its DNA-binding activity [116,117]. Oxidative modification of BZR1 at Cys63, which causes the cysteine thiol group (-SH) to be oxidized and generates a sulfenic acid group, enhances the transcriptional activity of BZR1 by promoting the interaction between BZR1 and the key regulators in the auxin-signaling and light-signaling pathways, including AUXIN RESPONSE FACTOR6 (ARF6) and PHYTOCHROME INTERACTING FACTOR4 (PIF4) [118]. Oxidative modification at Cys247 of soybean NAC WITH TRANS-MEMBRANE MOTIF1-LIKE 1 (GmNTL1) activates the expression of *RESPIRATORY BURST OXIDASE HOMOLOG B* (*GmRbohB*), *CATION H*+ *EXCHANGER 1* (*GmCHX1*)/*SALT TOLERANCE-ASSOCIATED GENE ON CHROMOSOME 3* (*GmSALT3*), and *Na*^+^/*H*^+^ *Antiporter 1* (*GmNHX1*) genes [119]. The rice zinc finger protein ZFP36 undergoes oxidative modification at Cys32, resulting in enhanced expression and activity of genes encoding protective antioxidant enzymes after abscisic acid (ABA) treatment [120]. Recently, it was found that oxidative modifications of a Cys residue in the transcription factor CCA1 HIKING EXPEDITION (CHE) promotes its binding to the promoter of the SA-biosynthesis gene *ISOCHORISMATE SYNTHASE1* (*ICS1*) [121]. Interestingly, the ROS sensors HPCA1 and PRXIIB, or the ROS production protein RBOHD and scavenging protein ascorbate peroxidase (APX1), also carry out S-nitrosylation [47,69,71,122]. During ovule development in *Arabidopsis thaliana*, H_2_O_2_ induces S-sulfenylation of cysteine at position 284 (Cys284) in the GSNOR1 protein, inhibiting its enzymatic activity. This inhibition subsequently leads to a decrease in the level of S-nitrosylated proteins (SNO) in the pistil, disruption of NO homeostasis, and ultimately causes ovule developmental defects [123].

Methionine (Met) is another amino acid that is susceptible to undergoing oxidation by ROS, which has received little attention in plants. The main types of oxidative modifications of Met include methionine sulfoxide (MetO) and methionine sulfone (MetO_2_) (Figure 2d) [124]. The biological significance of plant protein MetO modification remains largely uncharacterized, but a lot of evidence suggests that cyclic oxidation of Met is emerging as a mechanism by which proteins perceive oxidative stress and function in redox signaling. Tandem mass spectrometry (MS/MS) analysis shows that treatment with 10 μM or 50 μM of cyclic GMP (cGMP) or H_2_O_2_ enhances protein oxidation in *Arabidopsis* [125]. A proteome-wide study utilizing an in vivo protein-bound methionine oxidation assay identified over 500 sites of oxidation [126]. However, it was not until 2021 that the function of this modification in plants was reported. Methionine sulfoxidation of MaNAC42, MaEIL9, and SlMYC2 decrease their DNA-binding activity and transcription activity, as the modification sites are located near the conserved DNA-binding motif [127,128,129]. Recently, it has been reported that oxidized bHLH25 represses the expression of miR397b, leading to enhanced lignin biosynthesis. In contrast, non-oxidized bHLH25 promotes the expression of *Copalyl Diphosphate Synthase 2* (*CPS2*) [72]. These studies highlight the significance of methionine oxidation in regulating various aspects of plant physiology, including defense mechanisms and growth processes. However, further investigation is needed to fully understand the roles of methionine oxidation in plant defense responses and developmental pathways.

#### 4.3.2. Lipid-Peroxidation-Derived Reactive Carbonyl Species (RCS) Mediated Oxidative Modifications

ROS-triggered lipid oxidation causes production of highly reactive lipid peroxidation-derived molecules such as 4-hydroxy-2-nonenal (HNE), 4-hydroxy-2-hexenal (HHE), malondialdehyde (MDA), and acrolein. These unstable molecules are named as reactive carbonyl species (RCS) and high concentrations of RCS can cause irreversible damage in plant cells [130]. The addition of H_2_O_2_ to BY-2 cells caused increases in RCS such as acrolein, HNE, and HHE within 2 h. Addition of the chemical carbonyl scavengers carnosine and hydralazine suppressed H_2_O_2_-induced PCD symptoms in BY-2 cells [131]. It has also been reported that RCS is involved in salt-induced seed germination and root growth inhibition [132,133], low temperature [134], chilling [135], and hormone-dependent stomata closure [136,137]. However, the molecular mechanism by which RCS regulate plant responses to environmental stimulus remains to be explored.

RCS can react with specific target protein(s), which are referred to as protein carbonylation. Among the different amino acids, Cys, Lys, and His shows the highest rate of carbonylation by RCS [138,139]. Carbonyl-targeted proteins have been identified in salt-stressed *Arabidopsis* [140] and legume nodules [141]. Twenty-two oxidized proteins were detected in a mitochondrial matrix by 2D-LC-MS/MS after treatment with metal-catalyzed oxidation reagents CuSO_4_ and H_2_O_2_ [142]. Recently, 35 carbonylated proteins, including nitrate reductase NADH2 (NIA2), RNA-binding protein CP29B, and NADP-ME2, were specifically identified in exogenous H_2_O_2_-treated *Arabidopsis* [143]. However, the biological significance of this PTM in plant response to H_2_O_2_ is still quite unknown in plant systems.

#### 4.3.3. ROS-RNS-Mediated Protein Modifications

ROS and RNS are key players in the intricate network of oxidative stress processes [144,145]. Among RNS, nitric oxide (NO) is a primary component, synthesized by nitric oxide synthase (NOS) in various cell types. Emerging evidence suggests that NO plays a critical role in regulating ROS signaling pathways. On one hand, NO acts as an important endogenous mediator of H_2_O_2_. Studies have shown that heat shock-induced NO levels are reduced in atrbohB, atrbohD, and atrbohB/D plants, implying H_2_O_2_ acts upstream of NO in thermotolerance [146]. H_2_O_2_ has been shown to increase NO generation in maize leaf mesophyll cells. Pretreatments with NO scavengers cPTIO (2-phenyl-4,4,5,5- tetramethylimidazoline-1-oxyl 3-oxide) and l-NAME partly block H_2_O_2_-induced enhancements in the transcript levels of antioxidant genes and the activation of MAPK cascade [147]. Additionally, cPTIO significantly reduced H_2_O_2_-induced rice leaf cell death [148]. On the other hand, NO functions as a crucial signaling molecule by mediating protein S-nitrosylation, which provides feedback to the ROS signaling pathway. S-nitrosylation modulates ROS production associated with plant immunity. For instance, S-nitrosylation of the receptor-like cytoplasmic kinase (RLCK) botrytis-induced kinase 1 (BIK1) at Cys80 determines both the stability and activity of BIK1, thereby affecting flg22-induced ROS production [149]. Meanwhile, at Cys890 of NADPH oxidase, AtRBOHD, the substrate of BIK1, also undergoes S-nitrosylation, which abolishes the ability of AtRBOHD to synthesize reactive oxygen intermediates [46]. In the context of antioxidant processes, NO induces S-nitrosylation of the APX1 protein at Cys32, enhancing its enzymatic activity in scavenging ROS, maintaining cellular redox homeostasis, and ultimately improving the resistance to oxidative stress [122]. Collectively, ROS and RNS interact in the modulation of protein post-translational modifications.

## 5. Conclusions and Future Perspectives

This review delves deeply into aspects related to the generation and scavenging of ROS, ROS sensing, and ROS-mediated oxidative modifications. During the metabolic processes of plant cells, ROS can be generated via multiple pathways. These pathways encompass not only plasma-membrane-associated enzymes but also involve organelles such as chloroplasts and mitochondria. To maintain cellular redox homeostasis, plants have developed enzymatic and non-enzymatic antioxidant defense systems. Additionally, plants are capable of sensing intracellular and extracellular ROS through mechanisms involving HPCA1, QSOX1, or bHLH25, enabling them to respond effectively to environmental stimuli [69,71,72]. ROS can also mediate oxidative modifications in plants, affecting the function of DNA, RNA, lipids, and proteins. These modifications have significant implications for plant physiological and biochemical processes and play a crucial role in the interactions between plants and pathogens.

From this review, we conclude that a comprehensive understanding of oxidative modifications to nucleic acids, lipids, and proteins is essential. Additionally, several important questions warrant further investigation: (a) **Toxic molecules or spark of life**. In determining the outcomes of ROS production, what plays a crucial role: the ROS content, the targets of ROS, or the subcellular localization of ROS? (b) **The relationship between ROS production, ROS sensing, and oxidative modifications.** Do intracellular or extracellular ROS primarily regulate oxidative modifications? What role do ROS sensors play in these modifications? (a) **Site specificity determinants for oxidative modifications.** Given that proteins contain multiple cysteine (Cys) and methionine (Met) residues, how do these residues influence site specificity? (d) **Interplay between ROS, RNS, and RCS in oxidative modifications.** ROS, RNS, and RCS contribute to oxidative modifications of proteins at Cys residues, which enhances the complexity of cellular signaling. Investigating the factors that determine the type of oxidation—whether ROS, RNS, and RCS recognize different motifs and how one type of modification influences the others—will be essential for enhancing our understanding of oxidative modifications and their implications in plant cellular biology.

## Figures and Tables

**Table 1:** Oxidative post-translational modifications (oxiPTMs) regulating protein functions in plants.

	oxiPTM	Description
ROS	S-nitrosylation of Cys	NPR1 Cys: nuclear import and oligomerization
	S-sulfenylation of Cys	GSNOR1 Cys284: enzymatic activity inhibition
		BZR1 Cys63: transcriptional activity
	Oxidative modifications of Cys	GmNTL1 Cys247: activation of GmRbohB, GmCHX1/GmSALT3, GmNHX1 gene expression
		ZFP36 Cys32: enhanced expression and activity of antioxidant enzyme genes upon ABA treatment
		CHE Cys residue: promotes binding to ICS1 promoter
	Sulfoxidation of Met	Methionine sulfoxidation of MaNAC42, MaEIL9, and SlMYC2 decrease their DNA-binding activity and transcription activity
		bHLH25 Met256: transcriptional activity
RNS	S-nitrosylation of Cys	BIK1 Cys80: stability, activity, and flg22-induced ROS production.
		AtRBOHD Cys890: abolishment of reactive oxygen intermediates synthesis ability
		APX1 Cys32: ROS-scavenging activity
		MYB30 Cys49: transcriptional activity enhancement and PYL4 interaction disruption
